# Isovists compactness and stairs as predictors of evacuation route choice

**DOI:** 10.1038/s41598-023-29944-8

**Published:** 2023-02-20

**Authors:** Dajana Snopková, Laure De Cock, Vojtěch Juřík, Ondřej Kvarda, Martin Tancoš, Lukáš Herman, Petr Kubíček

**Affiliations:** 1 Department of Geography, Faculty of Science, Masaryk University, Brno, Czech Republic; 2 Department of Geography, Ghent University, Ghent, Belgium; 3 Department of Psychology, Faculty of Arts, Masaryk University, Brno, Czech Republic; 4 Interdisciplinary Research Team on Internet and Society, Faculty of Social Studies, Masaryk University, Brno, Czech Republic

**Keywords:** Human behaviour, Computer science

## Abstract

The building design is a crucial factor that can be actively adjusted and optimized to prevent human and property threats in emergency scenarios. Previous research suggests that specific building layouts may significantly influence human behaviour during evacuation. However, detailed empirical data about human behaviour in various types of buildings with different layouts are still missing and only marginal recommendations from this field are reflected in actual construction practice. In this study, desktop VR technologies were employed to study human decision-making in problematic T-intersections in the context of an emergency evacuation. More specifically, we studied fundamental attributes of buildings such as the width and length of the corridors and the presence of stairs to explore how they influence the choice of the evacuation route. The space-syntax isovist method was used to describe spatial parameters of corridors, which makes the results applicable to all buildings. Behavioural data from 208 respondents were analysed using multilevel regression models. Our results support previous claims concerning the importance of specific spatial layouts of evacuation corridors because respondents systematically chose wider and shorter corridors with visible staircases as the preferred evacuation route. The present findings further promote the ongoing discussion on the design of marked evacuation routes and building design that takes human factors into consideration.

## Introduction

Human evacuation behaviour in emergency situations is according to Proulx^1^ influenced by several factors such as cognitive and social aspects^2–5^ (e.g. personal characteristics, health, role^6,7^, knowledge, and previous experience^8–10^, condition at the time of the event^9,11^), the nature of specific hazards (natural disasters, gas leaks, terrorist attacks, shootings, fire^12^, etc.), and the situational and building design contexts (e.g. occupancy^13–15^, architecture and safety features^16^). It is not possible to conduct safety evacuation exercises with all potential visitors to a building to prepare them for possible hazards, nor can the type, origin, time of occurrence, or extent of these hazards be accurately predicted. Therefore, the building itself and its environmental design remain a major factor that can be actively adopted by architects, urbanists, and constructors in an effort to prevent human and property threats in emergency scenarios.

Building factors, as classified by Proulx^1^, cover occupancy (the type of the building based on its function), architecture (building layout), activities available in the building, and fire safety features (alarm, evacuation plan, and signage). At a glance, proper evacuation signage in a building appears to be the most instructive factor. Still, according to Wood^17^, people often fail to notice or ignore evacuation signs for various reasons, especially in reduced visibility situations or if the signage is not positioned appropriately^18^; therefore, it cannot be considered the ultimate evacuation aid. Nevertheless, following evacuation signs is the most common strategy in engineering practice such as computer-based evacuation simulations (agent modelling)^19^. Agents are often guided exclusively along marked evacuation routes^19^, and no other wayfinding strategies that are commonly used in the actual context are implemented, which contradicts real human behaviour^6,19^. For example, in real evacuation situations, people who are generally unfamiliar with the whole building layout try to return to the exit the same way they entered the building (even when the route is not marked), i.e. the so-called retracing strategy is used^1,6,10,20^. Based on these examples, evacuation behaviour is never completely straightforward and varies from individual to individual. Engineering practice, when assessing the safe capacity of buildings, focuses primarily on outliers (the slowest individuals), which increases the total evacuation time or, in the worst cases, casualties. Therefore, we assume that proper understanding and potential modelling of such outlying human decision-making and follow-up behaviour is crucial for reliable predictions of various evacuation situations.

According to the space syntax theory^21^, human decision-making occurring during navigation is influenced by the actual topology of a particular building^22^. Space syntax methods enable us to describe specific building layouts with the use of universally comparable quantified metrics. Navigation behaviour and strategies were found to correlate particularly well with these metrics. For example, the “least angle”^23–25^, “fewest turns”^26^, “follow their noses”^27^, “straight initial segment”^28^, and “central point” and “floor”^29^ strategies all trace their origin to the space syntax theory. The space syntax theory has also adopted the aspects of visibility that play a key role in decision-making^30,31^. Space visible from a certain point can be abstracted and projected in the form of 2D polygons called isovists^32^. Different isovist metrics (visual area, occlusivity, perimeter, compactness, etc.) have been proven to correlate with wayfinding performance^27,33–37^, with human comprehension of enclosed spaces^37,38^, and they can even subconsciously influence people's choices in everyday life^39^. The use of space syntax theory in the study of navigation has found applications mainly in everyday navigation and exploratory tasks. Only a few studies have applied knowledge from space syntax theory in the context of evacuation^10,40,41^.

It is difficult to conduct evacuation exercises for research purposes in existing buildings as it would interfere with their daily operations. Additionally, the parameters of existing buildings cannot be modified, although the best research strategy would be to test different spatial configurations. To this end, virtual reality (VR) provides the desired possibilities and functionality^42^. The use of VR technologies in behavioural research has increased^43^ in recent years, including the investigation of human behaviour and cognitive processes during the evacuation process^10,44,45^. VR tools represent promising instruments for studying poorly accessible or logistically complicated real-world scenarios^46^. Recently, the COVID-19 pandemic has made real-world research in this field even more challenging, and the need for remote data collection has increased considerably^47^. A current trend in research is the integration of VR and web technologies^48–50^, which allows researchers to perform behavioural experiments out-of-lab (i.e. outside the traditional controlled experimental environment). The web-based VR technology is still mainly associated with visualization on the computer monitor, so it is an example of desktop VR. In general, desktop VR is characterized by only a limited level of immersion^51^. However, desktop VR environments can still provide more realistic and ecologically valid conditions than traditional visualization methods such as maps, building plans, schemes, and graphs. It is not very demanding in terms of hardware and computing power since it is possible to use regular output devices. Therefore, it is easier to reach a desirable number of users/respondents. Web technologies also represent a suitable platform for an objective recording of respondents' activities (user logging), especially when they dynamically interact with a given product in their usual environment (at home)^52–54^. They provide a fruitful source of information and can bring insight into the decision-making processes and users' ways of thinking. This approach has been frequently used in recent decades in web design^55^ and mobile application evaluation^56^.

## Present study

In this research, human decision-making in problematic T-intersection corridors is studied during the process of an emergency evacuation. T-intersections were found to cause difficulties in active wayfinding performance, as well as during retrospective route and landmark identification^27^. Therefore, we chose them as the basic layout for our decision point. We further focused on how the presence of stairs, and changes in width and the length of the intersection corridors, affect human corridor choices. These parameters were chosen since they represent fundamental construction features of buildings and are independent of the building function in contrast to materials, textures, and decorations. The selected corridor parameters are generally present in every building around the globe. They represent basic parts of the construction plans or building information models (BIM) and, as opposed to decorations, surfaces or even evacuation signs, they are likely to remain unchanged after the construction of the building is finished^57^. At the same time, they serve as a suitable input for modelling evacuation scenarios, for example through agent models^19^.

From the psychological point of view, the *corridor width* is associated with the feeling of safety during emergency situations^40,58^ and privacy, when people are trying to maintain a comfortable distance from the walls and personal space from each other^59^. The corridor width has been addressed (together with brightness) by Vilar et al.^40^ as a significant predictor of evacuation behaviour. Wayfinders preferred wider (4 m and 3 m) and brighter corridors. When combining these factors, brightness increased the probability of choosing a wider corridor. Zhang and Park^57^ studied the influence of width, length, and height of corridors on the search for exits in underground malls, and also confirmed the wide corridor preference. Based on the study of Sun and de Vries^58^, wider exit doors are considered safer and are selected more often during evacuation. Also, a later study by Snopková et al.^10^ suggested that the increasing corridor width suppresses retracing tendencies, which is considered an unintentional wayfinding strategy during evacuation.

The *corridor length* can be related to curiosity and the desire to explore, where the longer corridors can provide more information than the shorter ones^60–62^. In game-like wayfinding tasks, respondents preferred longer corridors^61,62^; however during an evacuation, a well-known and anticipated strategy of choosing the shortest route prevails^57^.

The ratio of the corridor width and length can be expressed by the isovist^32^ *compactness* measure, which represents the shape of the space visible from a given location relative to the circle. Compactness ranges between 0 and 1 (circle) and is calculated by Eq. (1). The isovist metric compactness basically expresses the relationship between the width and length of the corridor. Sadalla and Oxley^63^ discovered an illusionary effect of rectangularity in room size estimations. Respondents considered rectangular rooms bigger than the square ones independent of the viewing position. A ratio of width and length was also used as a parameter in a space-syntax-based evacuation model for hospitals but only based on an assumption that "during an advantageous escape one needs to pass through cells shorter in distance but wider in extent" (Ünlü et al.^41^, p. 165). In explorative navigation tasks, Wiener et al.^37^ proved that points with high compactness values appeared to be more complex and it was more difficult for participants to identify the best hiding and overview places. De Cock et. al.^34^ also studied the influence of compactness on decision-making during turn-by-turn instructions guided navigation. They found that the influence of compactness was dependent on the type of decision point, while turns with high compactness induced more navigational complexity; conversely, this was the other way around for start and end points.

1 $$C_{v} = \frac{{4\pi A_{V} }}{{P_{V}^{2} }}$$

*C*_*V*_ compactness, *A*_*V*_ area (area of all space visible from a location), *P*_*V*_ perimeter (length of the edge of all space visible from a location).

According to existing literature, the third chosen building parameter—*the stairs*—can be classified in two ways. The stairs are an easily memorable functional navigational landmark^64^, even though there are some individuals who report disorientation after using them due to the rotation when moving vertically^65,66^. Montello and Pick^67^ presented evidence showing that in direction-pointing tasks, people have trouble mentally aligning floor plans of different levels in transition spaces (stairs, elevators). In terms of spatial relationships, stairs are also an important integration component connecting the individual floors, increasing the global and local integration values of connecting paths^66^. In terms of evacuation, the direction (up or down) of the staircase is also important. In Europe, the most common assembly points are located on the ground, in the case of high-rise buildings; evacuation from the roof is also possible, but this possibility can be limited in the event of high-rise fires, as was the case with the World Trade Center attack of 11 September 2001^6^. When designing stairs, it is essential to differentiate their appearance from the surroundings sufficiently, so that their function is clear at first glance. At the same time, their placement within the building should match the occupant's activity within the layout^65^.

Currently, there is no consensus on whether the route choice is also affected by its direction (whether it leads to the right or left), given the dominant hand used or the driving side of the road. Some studies suggest that there are tendencies to bear right^68^ (e.g. Robinson^69^; Scharine and McBeath^70^), but in other cases, no significant differences were found^40,71,72^.

Although these selected parameters have been already investigated in several studies^10,40,57,58,61,62,65,66,73^, a considerable research gap still persists. Many previous studies lacked the evacuation context^60–62^, or did not study these parameters systematically (e.g., Zhang & Park^57^ studied only a subset of all possible combinations of the given corridor length and width). No previous study was found that would deal with all the selected parameters and their interaction at the same time. From this perspective, this current experimental study represents a unique, systematic analysis of selected building parameters. In particular, the study is focused on their mutual interaction affecting human decision-making during evacuation. The study aims to promote the understanding of underlying cognitive and behavioural processes in T-intersections of indoor evacuation routes using up-to-date VR and web-based technologies. With respect to the above-discussed studies, we formulated several research questions:

- RQ1: Which are the strongest factors influencing corridor choices?
- RQ2: Does laterality influence corridor choices?
- RQ3: Which setup induces the shortest reaction time?
- RQ4: Which setup induces the highest level of confidence with the corridor choice?

The potential findings are meant to be applied in the engineering practice—that is in agent-based modelling of evacuation behaviour in buildings. In general, they should promote safety in the building design process.

## Methods

We conducted a within-subjects web-based online experiment, where each respondent received a random subset of 20 tasks. Each task presented a unique layout of the T-intersection in a building defined by the values of selected building parameters: corridor width, length, and presence of stairs. We measured the respondents' corridor choices, reaction time, and confidence in their responses.

### Respondents

The online study was in English and the participants included people from all over the world (the Czech Republic, Slovakia, Belgium, Philippines, Australia, etc.). A total number of 273 respondents completed the test, while 211 completed all tasks and completed the evaluation questionnaire. Two respondents had to be excluded due to technical issues with data logging. The decision-making of the respondents was limited in time, there was an 8-s timer at the bottom of the screen. Forty-eight cases where respondents did not manage to respond to the task in the given time were excluded from the analysis. We also excluded one respondent who missed the time limit for more than five tasks. None of the respondents was excluded due to visual impairment, not even people suffering from colour vision disorders, as the stimulus was sufficiently readable for them (tested with Coblis—Color Blindness Simulator^74^). Eventually, data from 208 respondents (F = 101, M = 107) aged 17 to 71 years (M = 31, MD = 26, SD = 12.36) were further analysed. Regarding education, more than half of the respondents (54%) had bachelor's or master's degrees; about 31% had completed primary or secondary education, and 14% had a doctorate or higher.

### Selected building parameters

The following building parameters were chosen for this study: *width* (2 and 4 m), *corridor length* (10, 15, and 20 m), and the presence of *stairs* (0—corridor without stairs/1—corridor with stairs) in the respondents' field of view. The width of the stairs varied as well and was determined by the changing width of the corridors. The combination of corridor width and length can also be expressed through isovist (space-syntax) metrics, see Table 1. Specific corridor width values have been set with regard to the study of Vilar et al.^40^. They used minimum width of corridors 2 m set according to the Portuguese Technical Regulation for Fire Safety in Buildings from 2008 and 2.5; 3; 3.5 and 4 m width for comparison. The study proved similar attractivity of 3 and 4-m wide corridors, therefore the final width choice for our study was 2 and 4 m. Specific values of corridor length were chosen, ensuring a continuous range of the resulting isovist compactness values.

**Table 1:** Isovist metrics calculated for different corridor width and length combinations.

Corridor width	Corridor length	Isovist metrics		
		Area	Perimeter	Compactness
2	10	22.83	28.01	0.37
2	15	30.77	35.77	0.30
2	20	42.96	46.77	0.25
4	10	41.64	28.14	0.66
4	15	60.85	37.55	0.54
4	20	81.06	46.89	0.46

The third influencing factor was whether the particular combination of parameters was on the *right or the left* from the evacuee's point of view. It was most appropriate to assess the impact of these parameters at a T-intersection where evacuees only had two corridor options to choose from (assuming they did not want to return the way they came). By combining all the selected factors, a total of *63 unique intersection layouts* were generated, see Supplementary Figure 1.

### Stimuli

**Figure 1:**
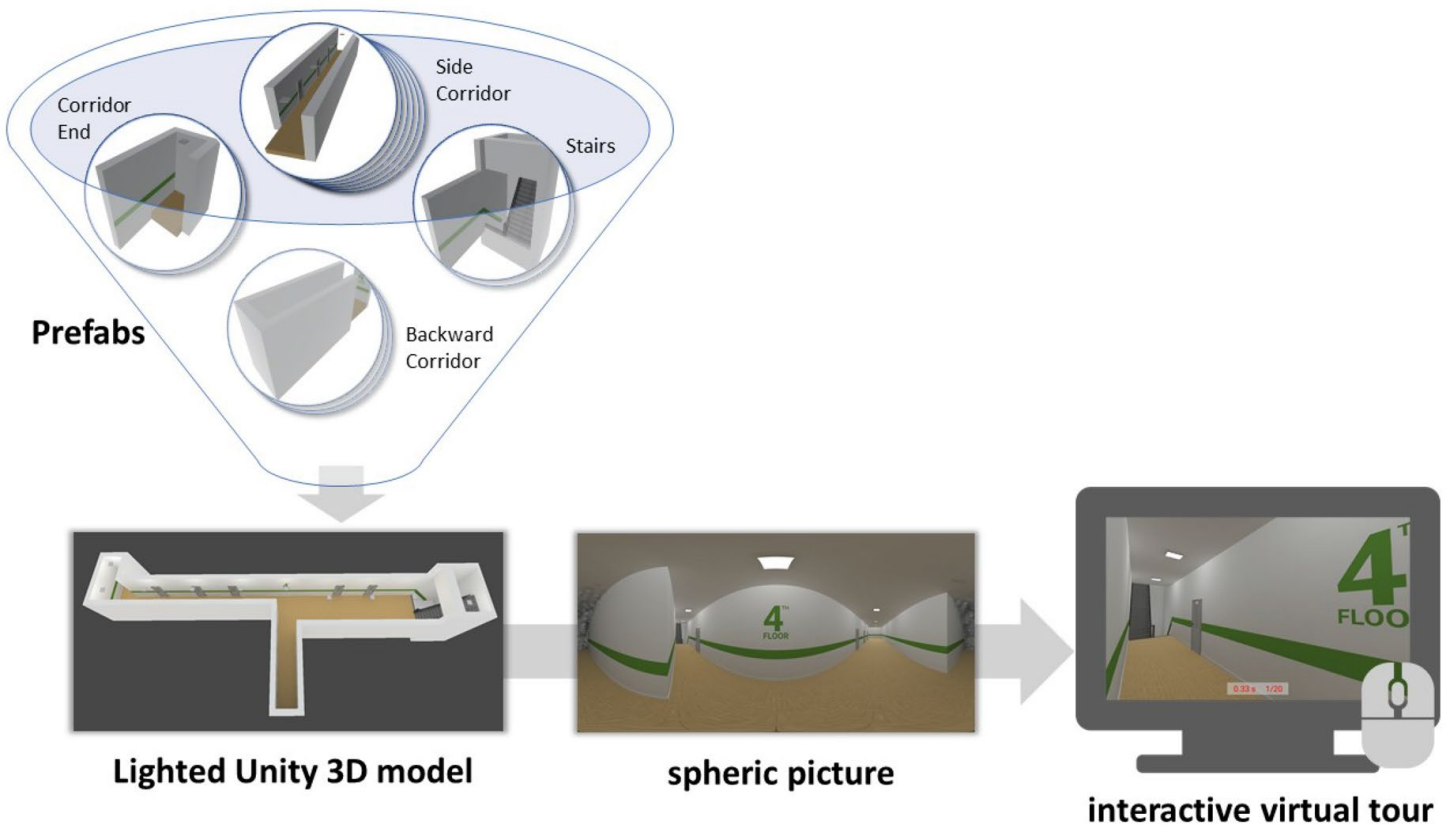
Stimuli creation process.

3D models of individual intersection variants were created using the Unity game engine's URP (Universal Render Pipeline). Each variable (different widths/lengths of the corridors, widths of the "start points" and endpoints of corridors, and widths of the staircases) was modelled independently on the rest (as a prefabricated object group—a Prefab) to be then easily assembled into the desired layouts, much like Lego bricks, see Fig. 1. This versatility proved beneficial with regard to applying textures to objects and several experiment design changes when only the Prefab had to be manually edited for the change to be reflected in all combinations. After assembly, these individual combinations were lit using the precomputed Baked GI (Global Illumination) Lighting, which calculates light effects on static objects and then writes the results into (lightmap) textures that are overlaid on top of the objects giving the impression of artificial lighting.

Since the individual intersections were not presented to respondents via immersive virtual reality but in the form of a web-based virtual tour, all corridor combinations had to be exported in the form of spheric pictures, see Fig. 1. These were taken in runtime by the Unity 360° Screenshot Capture plugin^75^ and exported as PNG (Portable Network Graphics) files with 4098 × 4098 pixel resolution. Due to the loading time in the browser, it was necessary to optimize the size of individual scenes further. So the scenes were compressed with the pngquant tool v. 2.17.0^76^ and saved in JPG (Joint Photographic Experts Group) format. For the web presentation of the created visual stimuli, we used the JavaScript framework A-Frame 1.2.0^77^, which allows displaying 3D models, 360° images, and videos for mobile and desktop devices, as well as special VR and AR devices. It also provides native controls with additional customization options. The entire web application was programmed with a combination of HTML (Hypertext Markup Language), CSS (Cascading Style Sheets), JavaScript, PHP (Hypertext Preprocessor), and Bootstrap 5.1.1. library^78^ and is publicly available on GitHub (https://github.com/VGE-lab-MUNI/evacuation-indoor-experiment/import). The resulting displaying device used during the experiment depended on each respondent's possibilities, given the nature of the online study. However, in the background, we collected information about the screen resolution and whether the respondents worked in full-screen mode, which made it possible to exclude unsatisfactory cases.

### Design, procedure and data collection

**Figure 2:**
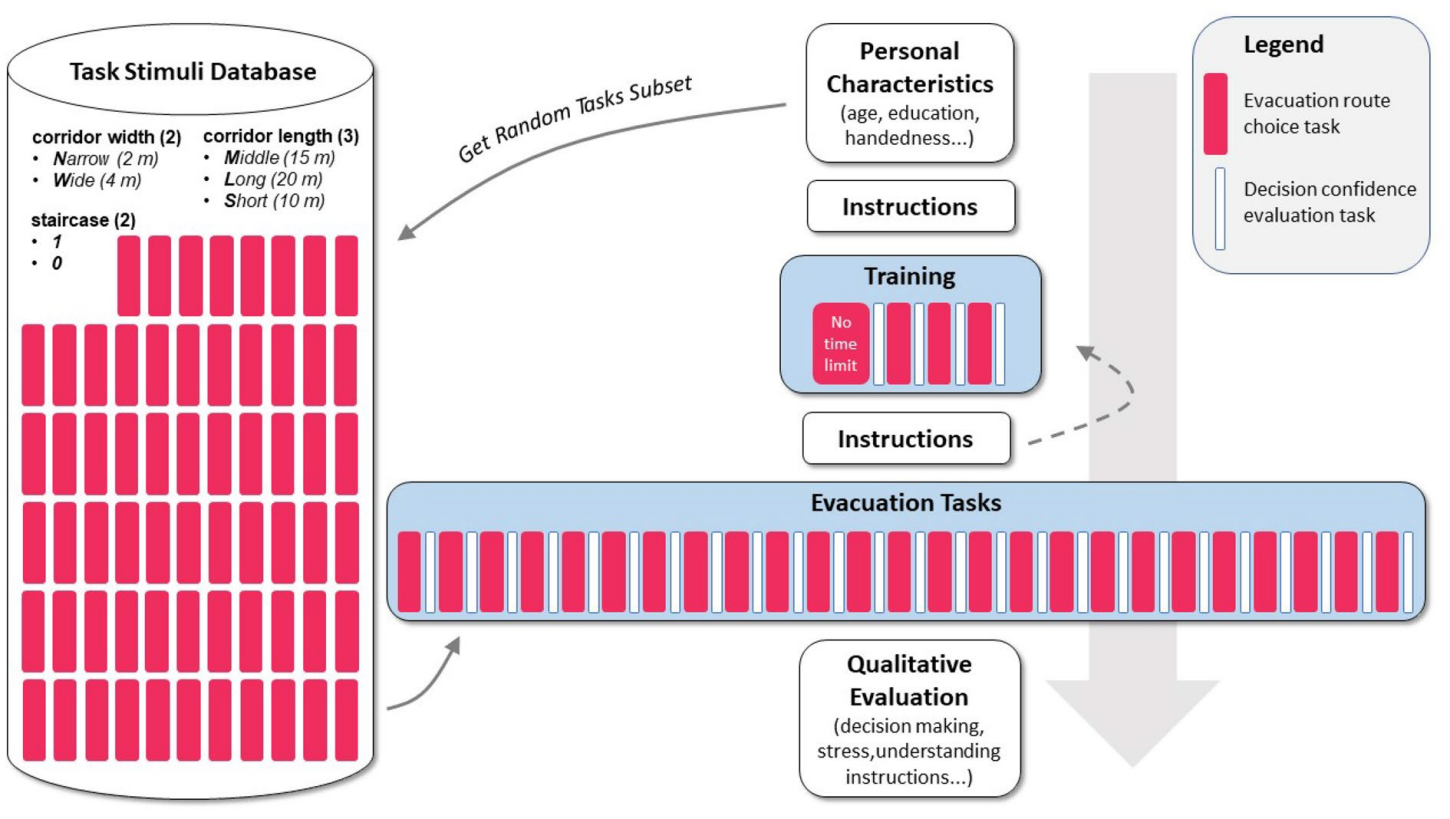
Experiment procedure.

The whole experimental procedure is illustrated in Fig. 2. The study was assessed and approved as ethically indisputable by the Ethics board under the Department of Psychology of the Faculty of Arts, Masaryk University, Brno. The study was conducted in accordance with relevant guidelines and regulations following the principles of Declaration of Helsinki. Even though this online study was completely anonymous, informed consent with all information about the experiment was presented to be read and confirmed by all respondents before the start of the experimental session. The introductory page of the online study additionally included a brief introduction to the experiment and a description of the data to be collected. Next, a questionnaire of *personal characteristics* (age, gender, educational attainment, left- or right-handedness, driving side of the route, eye defects) followed, in which a unique identifier was generated for each respondent. Afterwards, the respondents received *instructions* informing them that they were in a building where a fire had started, and they ran out of the office and found themselves at an intersection where they had to decide where to go. The site also contained a description of the scenes' controls, followed by a display of a scene where the respondents could try the controls (without a time limit). Three *training questions* followed thereafter. At the end of the training, respondents had the opportunity to return to the instructions or continue with the test. The instructions were repeated. The study was administered using a web-based test, which is available at: https://olli.wz.cz/webtest/evacuation-indoor-experiment/. The recording of a sample experiment run is available in Supplementary Video 1.

A *within-subjects multifactorial design* was applied where each respondent received a random subset of 20 tasks from a full battery of 63 tasks. The respondent had 8 s for each decision; a countdown was displayed at the bottom of the page to simulate stress conditions. After selecting a corridor, a Likert scale appeared, on which the respondent was asked to record his or her *confidence with the given choice*. Then another task followed. If the respondent did not manage to select any corridor within 8 s, a message informed them that the time limit had expired, and the next task followed. No replacement task was given. After going through all 20 tasks, an evaluation questionnaire followed by measuring a *subjective opinion* on the *significance of individual factors* (5-point Likert scale: strongly disagree—strongly agree), the level of perceived stress, the clarity of instructions, and a mandatory open question where the respondents were asked to describe their decision-making strategy.

**Figure 3:**
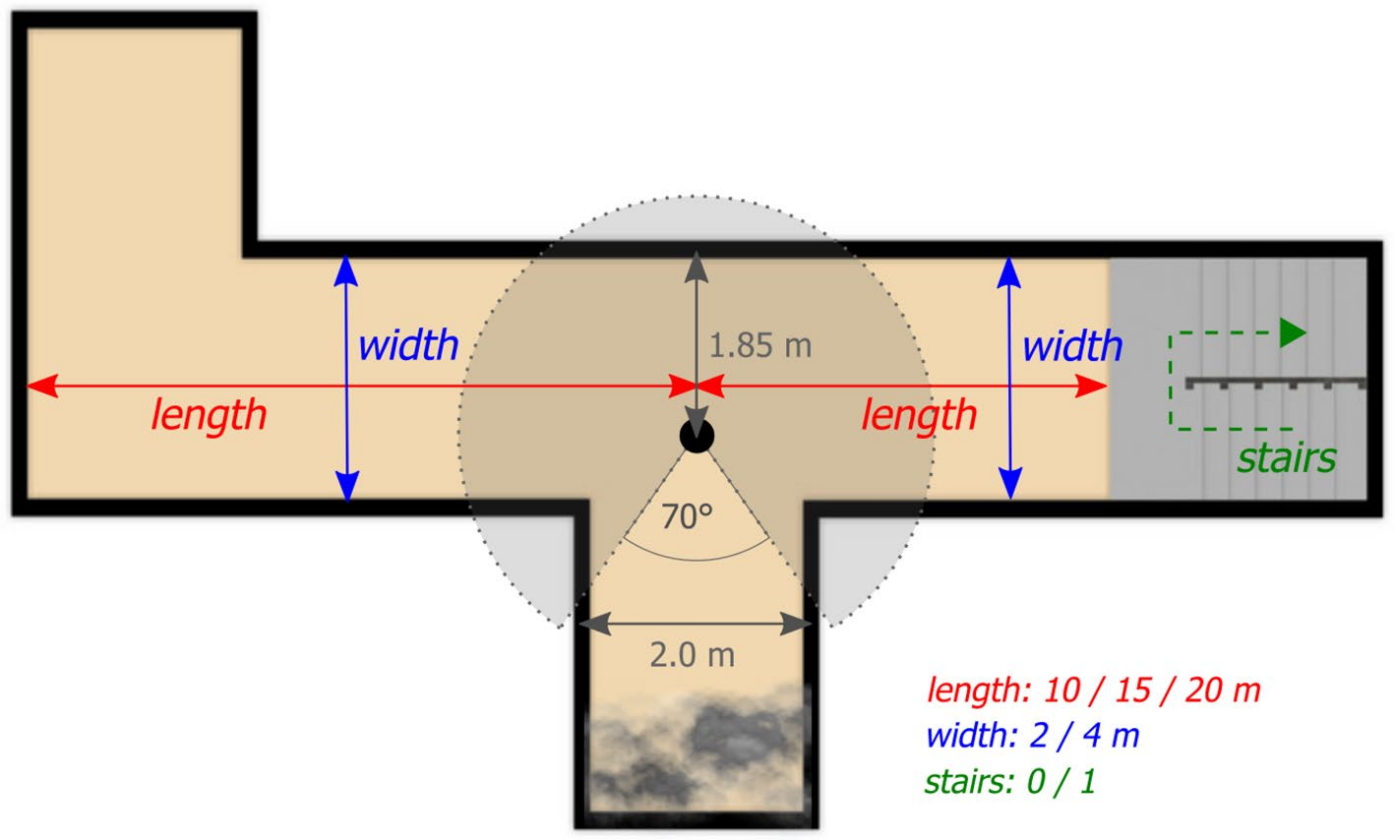
A sample decision point layout with an explanation of changing experimental variables.

The individual scenes were presented to the respondents through an *interactive spheric picture*. The respondents could not move; they could only *rotate* their field of view in the range of 290° (head rotation 170° + binocular visual field of view 120°). On each task initiation, the respondents were turned to face the wall, did not see the evacuation corridors, and thus were forced to interact with the panorama. In addition, a smoke heap was placed behind their backs, indicating the source and direction of the hazard. Figure 3 illustrates the layout of the intersection within which the respondents made their decisions. The layout remained fixed throughout the whole experiment; only the selected building parameters varied. Respondents used only one input device to interact with the scene—a computer mouse for rotation and selecting a corridor (mouse-click). In the background, we collected quantitative data on user interaction with the scene for each decision. Specifically, we collected *decision times* (times that took them to select the corridor), and the *selected corridor* (mouse click on invisible area of interest (AOI); left or right).

### Statistical analysis

The study represents a within-subjects multifactorial design including 3 factors (lengths and widths of the corridors, and the presence of a staircase in the corridor). Data were analysed in the R programming language^79^ using logistic multilevel regression models (packages lme4^80^ and lmerTest^81^) since the outcome variable of corridor selection is binary and the task data are nested in every participant. Reported *p-*values were obtained using Satterthwaite approximation^82^ for degrees of freedom. Confidence intervals were calculated by the percentile bootstrapping method using 5000 simulations.

In the analysis process, two separate types of hierarchical models were built. The first, designated Models 1A and 1B, included the predictors of length, width, and the presence of stairs predicting the binary variable of the selected corridor. The second, designated Models 2A and 2B, replaced the length and width parameters with the isovist metric of compactness, covering the information of length and width in a more general (scale-independent) parameter. Both model types were built in three steps, the first one—the null model—included only two uncorrelated random intercept terms for respondents and specific tasks. The second step included the predictors characteristic for both model types. Above that, the third step also included the random slope terms for respondents in width and length (in model 1B) or in compactness (in model 2B). Due to convergence difficulties, all mentioned random terms were specified as uncorrelated. This means that random term vectors of estimated values for every person (cluster) are independent from each other (e.g., a person having higher random intercept value does not have higher probability of having any higher random slope value and vice versa). Random term variances stand as a separated variance components with no shared variance. Model equations are listed below:

2 $$\begin{aligned} & {\mathbf{Model}}\;{\mathbf{0}} \\ & {\text{logit}}\left( {corridor_{ij} } \right) = \beta_{ij} + \varepsilon_{ij} \\ & \beta_{ij} = \gamma_{00} + u_{0j} + u_{i0} \\ \end{aligned}$$

3 $$\begin{aligned} & {\mathbf{Model}}\;{\mathbf{1B}} \\ & {\text{logit}}\left( {corridor_{ij} } \right) = \beta_{ij} + \beta_{1j} *width_{{4{\text{vs}}.{ }2}} + \beta_{2j} *length_{{15{\text{vs}}.{ }10}} + \beta_{3j} *length_{{20{\text{vs}}.{ }10}} + \beta_{4j} *stairs + \varepsilon_{ij} \\ & \beta_{ij} = \gamma_{00} + u_{0j} + u_{i0} \\ & \beta_{1j} = \gamma_{10} + u_{1j} \\ & \beta_{2j} = \gamma_{20} + u_{2j} \\ & \beta_{3j} = \gamma_{30} + u_{3j} \\ & \beta_{4j} = \gamma_{40} \\ \end{aligned}$$

4 $$\begin{aligned} & {\mathbf{Model}}\;{\mathbf{2B}} \\ & {\text{logit}}\left( {corridor_{ij} } \right) = \beta_{ij} + \beta_{1j} *compactness + \beta_{2j} *stairs + \varepsilon_{ij} \\ & \beta_{ij} = \gamma_{00} + u_{0j} + u_{i0} \\ & \beta_{1j} = \gamma_{10} + u_{1j} \\ & \beta_{2j} = \gamma_{20} \\ \end{aligned}$$

$\mathrm{logit}()$ logit link function of the dependent variable (logarithm of the odds), ${corridor}_{ij}$ corridor selection in task *i* for person *j*, ${\beta }_{ij}$ intercept of corridor selection in task *i* for person *j*, ${\beta }_{1j}$…${\beta }_{4j}$ slopes of dependent variables for person *j*, ${\gamma }_{00}$ fixed intercept of corridor selection, ${\gamma }_{10}$…${\gamma }_{40}$ fixed slopes of dependent variables, ${u}_{0j}$ random intercept of corridor selection for person *j*, ${u}_{i0}$ random intercept of corridor selection for task *i*, ${u}_{1j}$…${u}_{3j}$ random slopes of dependent variables for person *j*, ${\varepsilon }_{ij}$ random errors of prediction in task *i* for person *j*.

### Bradley–Terry data transformation

Since the evacuation tasks were created as dichotomous decisions between left and right corridors with assigned parameters, the difference between both corridors in each specific task had to be considered. In order to meet this condition, we transformed the data according to the Bradley–Terry model^83,84^, which was developed exactly for the outcomes of paired comparisons. These transformations have some advantages, which make them suitable to answer our research questions: they allow us to directly compare the preference of one selected corridor with certain parameters against an unselected corridor, and at the same time the regression coefficients express the preference of each corridor characteristic regardless of whether the corridor was on the left or on the right side in a specific task.

Specific dummy variables were created with respect to length, width, and the presence of stairs. The transformation key for every type of possible corridor combination is listed in Table 2. Generally, if both corridors share the same level of a specific variable, the value of the dummy variable is 0. If the right corridor has a higher value of a specific variable, the dummy variable value is + 1; if the left corridor has a higher value of a specific variable, the dummy variable value is − 1. In the case of compactness, the values for the right corridor in each task were simply subtracted from values for the left corridor. A positive value indicates higher compactness in the right corridor, while a negative value indicates higher compactness in the left corridor.

**Table 2:** Dummy variable coding using the Bradley–Terry model.

Corridor width			Corridor length				Presence of stairs		
					Variable values				
Left	Right	Values	Left	Right	15 versus 10	20 versus 10	Left	Right	Values
2	4	+ 1	10	15	+ 1	0	0	1	+ 1
4	2	− 1	10	20	0	+ 1	1	0	− 1
2	2	0	15	10	− 1	0	0	0	0
4	4	0	15	15	0	0			
			15	20	− 1	+ 1			
			20	10	0	− 1			
			20	15	+ 1	− 1			

## Results

We first report the distribution of respondents' corridor choices (*RQ1*), reaction times (*RQ3*), and confidence in responses (*RQ4*) considering individual corridor parameters (the presence of stairs, corridor width and length). Next, we report the results of the follow-up questionnaire investigating the respondents' personal preferences for individual building factors and the course of the online experiment. The respondents’ corridor choices were further analysed by means of multilevel regression models (*RQ2*) after applying the Bradley–Terry transformation^83^.

**Figure 4:**
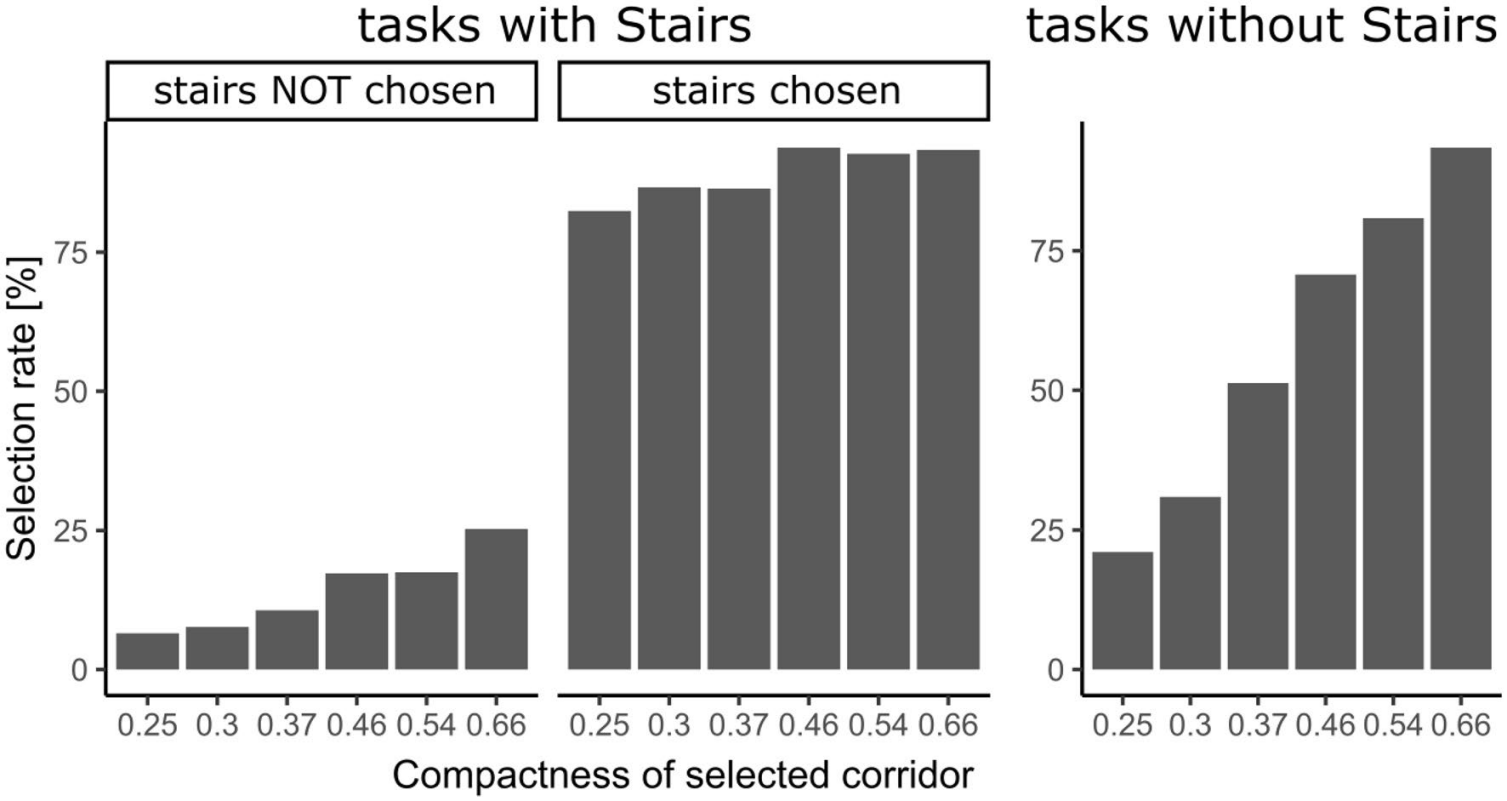
Distribution of respondents' selection rate grouped by task type, presence of stairs and compactness.

Due to task subset randomization, we obtained different number of answers per each task (MIN = 55, MAX = 80, M = 66, MED = 66, SD = 6.23). Each task always consisted of two corridors—two available choices, each specified by values of three selected building parameters. Supplementary Table 1 shows the number of times the given corridor has been selected and concurrently not selected. Since each corridor setup appeared in the task stimuli database a different number of times, we normalized the data and calculated the "selection rate" as a ratio of the selected count to the sum of selected and non-selected counts. The highest selection rate (ranging from 82.43 to 93.82) was observed when the T-intersection contained a corridor with a staircase (*RQ1*). In both task types, the selection rate was higher for corridors with high isovists compactness values (0.66) compared to low isovists compactness values, see Fig. 4.

**Figure 5:**
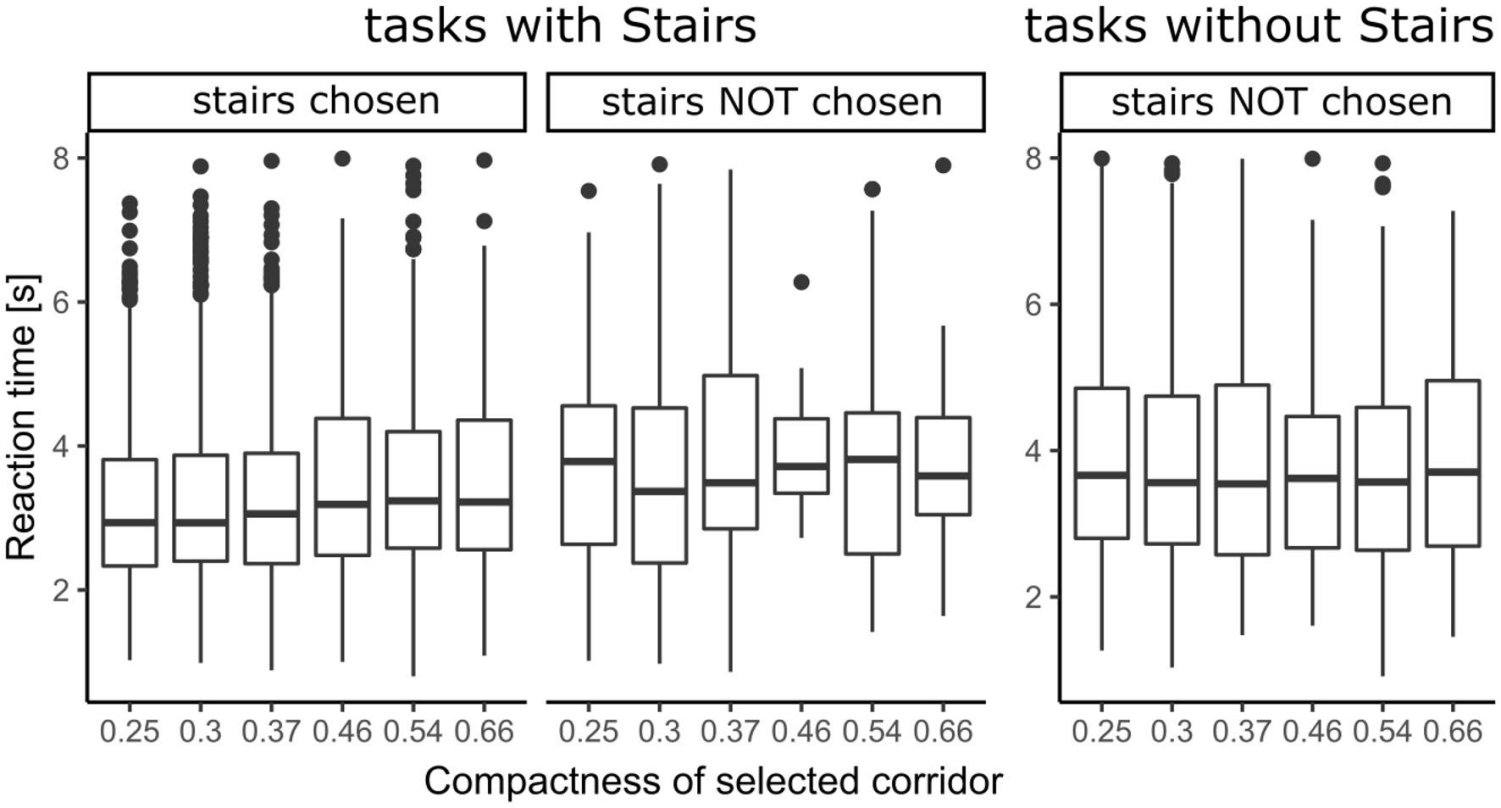
Distribution of respondents' reaction times grouped by task type, presence of stairs and compactness *(box corresponds to the interquartile range with a marked median, whiskers are computed as largest (smallest) value within 1.5 times interquartile range above 75th (below 25th) percentile, outliers are defined as values > 1.5 times and < 3 times the interquartile range beyond either end of the box).*

The decision-making of the respondents was restricted to 8 s for each T-intersection. On average it took respondents 3.53 s (s) to make their choice (MIN = 0.80, MAX = 7.99, M = 3.25, SD = 1.37). In general, respondents decided faster in intersections with stairs (*RQ3*, MEAN ranging from 2.91 s to 3.61 s for various corridor compactness values), and slowest (MEAN 4.24, 4.26 s) in intersections without stairs or containing corridors with low isovists compactness values (0.25, 0.30), see Fig. 5 and Supplementary Table 1.

After each task, respondents evaluated on the Likert scale (1—very unsure, 5—very confident) how confident they felt with their answers. In average respondents felt neutral (MEAN = 3.49, M = 4, SD = 1.00). Respondents reported the lowest levels of confidence (MEAN ranging from 2.61 to 3.15) in tasks without stairs and the highest in T-intersections with a staircase (*RQ4*, MEAN ranging from 3.66 to 3.98).

**Figure 6:**
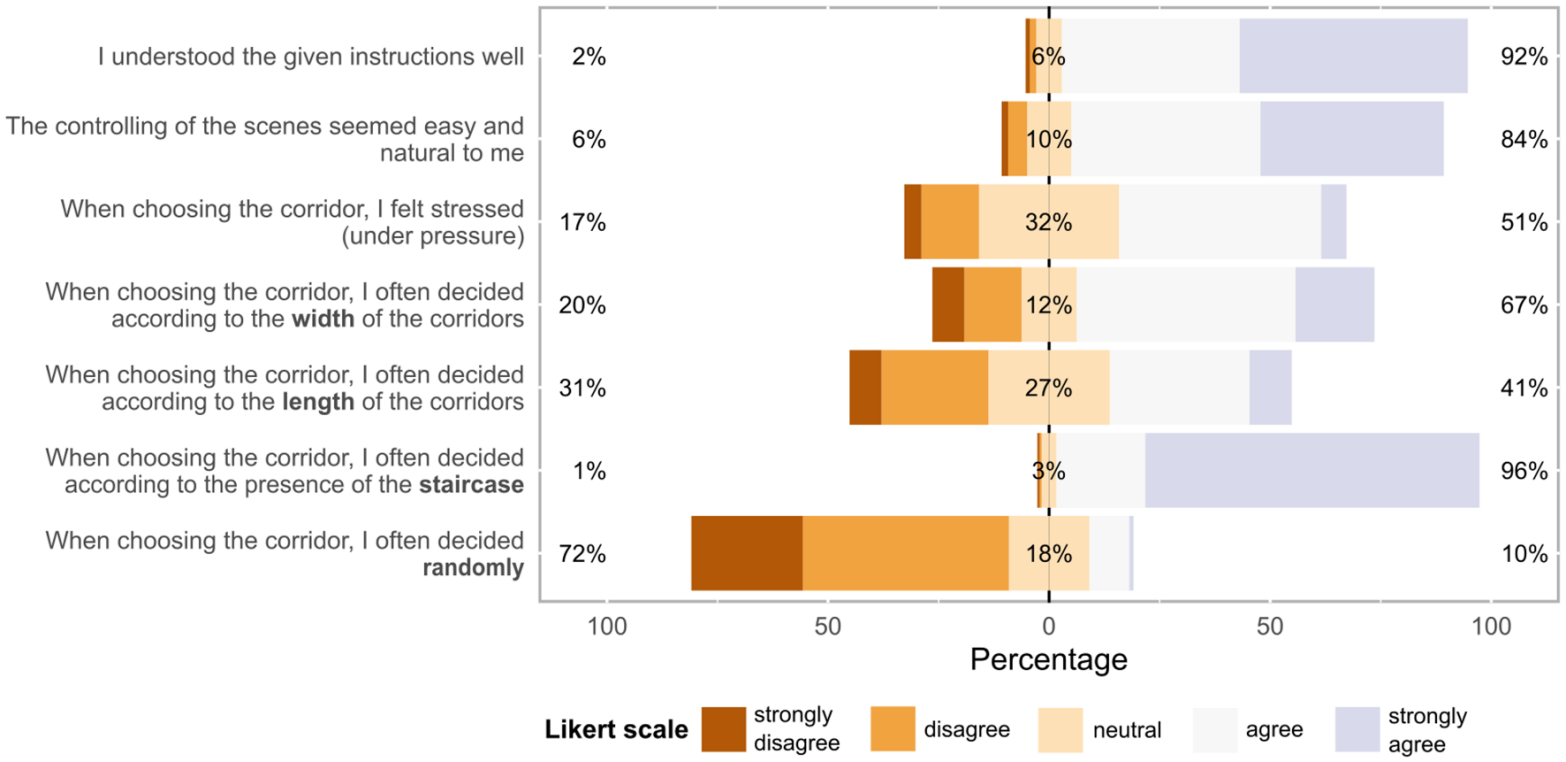
Summary of respondent responses to the electronic questionnaire.

According to the results from the closing questionnaire illustrated in Fig. 6, the experiment instructions were understandable for the respondents, and the scene controls were easy to use. We also managed to simulate stress conditions. Only 10% of respondents stated that they decided randomly; the strongest decision-making building factor was the stairs, followed by the width and length of the corridors.

### Bradley–Terry models

The null model with only random intercept terms for respondents and evacuation tasks showed that respondents did not prefer systematically left or right corridors (*RQ2*) regardless of their parameters since the fixed intercept is not significant, *b* =  − 0.01, *p* = 0.983, *OR* = 0.99, 95% CI (0.58, 1.67). Random intercept terms also showed that there was not a large variability in that preference between the respondents, σ^2^ = 0.12, 95% CI (0.03, 0.22). However, variability between the tasks in left or right corridor preference was much higher, σ^2^ = 4.44, 95% CI (2.81, 6.26). Thus, one of the corridors in a specific task had a higher probability of being chosen than the opposite one.

The results of Models 1A and 1B are provided in Table 3. Fixed effects of Model 1A showed that there was a significant difference in the probability of choosing wider corridors in comparison to the narrow ones, *b* = 1.04, *p* < 0.001, *OR* = 2.82, 95% CI (2.35, 3.41). The odds of selecting wider corridors were 2.82 times higher. As regards the length parameter, the shortest length (10) was selected as a reference category. Respondents had significantly lower probability of either selecting the corridor with medium length (15),* b* =  − 0.37, *p* = 0.001, *OR* = 0.69, 95% CI (0.55, 0.86); or the longest length (20), *b* =  − 0.55, *p* < 0.001, *OR* = 0.57, 95% CI (0.46, 0.72). Negligible differences between both effect sizes also suggest that respondents did not really differentiate between medium and the longest corridors; the only meaningful difference is choosing against the shortest corridors. Results also showed that respondents had a very strong preference for stairs since the odds of selecting a corridor with stairs were 9.70 times higher than selecting a corridor without stairs, *b* = 2.27, *p* < 0.001, *OR* = 9.70, 95% CI (7.94, 11.91).

Random intercept variance for respondents stays at the same value as in the null model, σ^2^ = 0.13, 95% CI (0.03, 0.23). On the other hand, random intercept variance for tasks is substantially lower, since the fixed effects of corridor parameters took over some of the variability in tasks, σ^2^ = 0.20, 95% CI (0.07, 0.29). Model 1A had also a significantly better fit than the null model, Δχ^2^(4) = 155.9, *p* < 0.001; as seen in Table 5.

Model 1B included random slope terms for respondents in width and length above the parameters in Model 1A. Due to convergence issues, the random slope for the difference between medium (15) and (10) length was dropped. Only the term for the difference between long (20) and short (10) length remained. The individual differences in preference for wider corridors were quite high, σ^2^ = 1.69, 95% CI (1.10, 2.18), and the individual differences in preference for long corridors were considerably lower, σ^2^ = 0.42, 95% CI (0.12, 0.63). Fixed effects remained generally unchanged, one exception consisted of the suppressed effect of the presence of stairs, *b* = 2.70, *p* < 0.001, *OR* = 14.97, 95% CI (11.30, 19.04). Model 1B has a significantly better fit than Model 1A, Δχ^2^(2) = 184.5, *p* < 0.001 (see Table 5). Thus, random slope terms provided enough new information.

**Table 3:** Results of logistic models with separated corridor parameters.

	Model 1A					Model 1B				
Fixed effects				95% CI *OR*					95% CI *OR*	
	*b*	*p*	*OR*	LL	UL	*b*	*p*	*OR*	LL	UL
Intercept	0.03	.691	1.03	0.89	1.20	0.04	.695	1.04	0.86	1.25
Width 4 versus 2	1.04	< .001	2.82	2.35	3.41	1.25	< .001	3.50	2.60	4.59
Length 15 versus 10	− 0.37	.001	0.69	0.55	0.86	− 0.43	.002	0.65	0.49	0.87
Length 20 versus 10	− 0.55	< .001	0.57	0.46	0.72	− 0.66	< .001	0.51	0.39	0.70
Stairs	2.27	< .001	9.70	7.94	11.91	2.70	< .001	14.97	11.30	19.04

Random effects				95% CI σ^2^					95% CI σ^2^	
	σ^2^			LL	UL	σ^2^			LL	UL
RI respondents	0.13			0.03	0.23	0.17			0.02	0.28
RI tasks	0.20			0.07	0.29	0.34			0.12	0.45
RS width 4 versus 2						1.69			1.10	2.18
RS length 15 versus 10						–			–	–
RS length 20 versus 10						0.42			0.12	0.63

RI, random intercept; RS, random slope.

The width and length of the corridors can also be expressed as compactness using the isovist method. This derived metric was used in follow-up Models (2A and 2B), the results of which are shown in Table 4. Fixed effects of Model 2A found that respondents are significantly more likely to choose corridors with higher compactness, *b* = 0.41, *p* < 0.001, *OR* = 1.51, 95% CI (1.41, 1.62). This means that every 0.1 increment in corridor compactness value increases the odds of selecting that corridor by a factor of 1.51. The strong preference for corridors with stairs remained unchanged in comparison with Model 1A, *b* = 2.26, *p* < 0.001, *OR* = 9.66, 95% CI (7.87, 11.83), same as the random intercept variances for respondents, σ^2^ = 0.13, 95% CI (0.03, 0.23); and tasks, σ^2^ = 0.21, 95% CI (0.09, 0.33). Model 2A also had a significantly better fit than the null model, Δχ^2^(2) = 153.8, *p* < 0.001 (see Table 5).

Model 2B included the random slope term for respondents in compactness above the parameters in Model 2A. The individual differences between the respondents in preference for different levels of compactness were large, σ^2^ = 20.18, 95% CI (12.83, 26.84). The fixed effects were again generally of the same values, the random slope terms slightly suppressed the effect of stair presence in Model 2B as well, *b* = 2.58, *p* < 0.001, *OR* = 13.14, 95% CI (10.03, 16.71). Model 2B had a significantly better fit than Model 2A, Δχ^2^(1) = 131.8, *p* < 0.001 (see Table 5).

**Table 4:** Results of logistic models with compactness parameter.

	Model 2A					Model 2B				
Fixed effects				95% CI *OR*					95% CI *OR*	
	*b*	*p*	*OR*	LL	UL	*b*	*p*	*OR*	LL	UL
Intercept	0.03	.720	1.03	0.88	1.20	0.03	.734	1.03	0.86	1.26
Compactness (0.1 unit)	0.41	< .001	1.51	1.41	1.62	0.50	< .001	1.64	1.48	1.83
Stairs	2.26	< .001	9.66	7.87	11.83	2.58	< .001	13.14	10.03	16.71

Random effects				95% CI σ^2^					95% CI σ^2^	
	σ^2^			LL	UL	σ^2^			LL	UL
RI respondents	0.13			0.03	0.23	0.17			0.03	0.27
RI tasks	0.21			0.09	0.33	0.37			0.15	0.52
RS compactness						20.18			12.83	26.84

The models with length and width could be perceived as competing with compactness models, as they try to provide similar information about the corridor space. Thus, the information criteria of these models were also compared, taking model complexity into account, see Table 5. Comparing only the random intercept Models (1A and 2A), they were almost equal in these metrics. The model with compactness (2A; AIC = 3383.7, BIC = 3415.3) slightly outperformed the model with length and width (1A; AIC = 3385.7, BIC = 3428.9). The situation changes when including random slopes, the compactness model (2B; AIC = 3253.9, BIC = 3291.8) is clearly outperformed by the model with length and width (1B; AIC = 3205.2, BIC = 3262.1). Moreover, Model 1B (bold in Table 5) turned out to be the best model according to the information criteria, even though it is the least stable when estimated. Thus, if we consider individual differences in corridor preference, the model including length and width parameters fits the data better. As regards simpler models, both views on corridor preference (width and length vs. compactness) are equally informative and could be used more or less interchangeably.

**Table 5:** Regression model fit indices.

	LL	AIC	BIC	Δχ^2^	*df*	*p*
Model 0	− 1763.8	3533.6	3552.5			
Model 1A	− 1685.8	3385.7	3429.9	155.9	4	< .001
Model 1B	** − 1593.6**	**3205.2**	**3262.1**	184.5	2	< .001
Model 2A*	− 1686.9	3383.7	3415.3	153.8	2	< .001
Model 2B	− 1621.0	3253.9	3291.8	131.8	1	< .001

*N,* 208, *obs,* 4113; LL, log − likelihood; *, χ^2^ compared to null model.

## Discussion and conclusion

In this study, we explored the influence of various aspects of indoor building design on the human decision-making process during the evacuation from a building. Using a web-based VR experiment, respondents were presented with a T-intersection where they had to decide which corridor (from the two available) they would use in case of evacuation from a building. Three selected building parameters (corridor width, length, and presence of the stairs) were modified in each of the intersection stimuli. Based on the analysis of gathered empirical data from 208 respondents, we employed multilevel regression models with Bradley–Terry transformation to identify several significant trends in human evacuation behaviour, which are discussed below.

First, the study findings do not suggest that respondents systematically prefer left or right corridors regardless of their other parameters, which corresponds to the prevailing opinion about no specific direction preference among evacuees^40,71,72^. The above-discussed notions from older studies^55,69^ about right direction relevancy were not supported, at least in this VR-based experimental session. This finding is also important in the context of this study since it allows us to make clearer predictions about other selected factors of the building when using statistical models. For the analysis of the data, we used two different sets of multilevel logistic regression models, one considering width, and length of the corridors as two separate factors, and the other engaging a calculated isovist metric—compactness, which indicates a ratio of width and length of the corridor. These modelling approaches could be perceived as competing as they try to provide similar information about the corridor space, so the information criteria of these models were compared, taking model complexity into account. According to the conducted analyses the models which did not take into account individual differences were identified as interchangeable. If we included random effect for participants, the model with corridor length and width outperformed the model with compactness. However, the advantage of the model using compactness is that this metric values are relative and, therefore, the results are applicable to all buildings corridors and not only to corridors with the exact widths and lengths that were used in this study. We report and discuss both of these approaches separately to provide a complete perspective on the data, and then we derive specific conclusions.

Applied models considering width and length as separate factors consistently showed a higher tendency among respondents to choose wider corridors to egress the building, where the probability of choosing wider as opposed to narrow corridors was approximately three times higher. This observation supports previous findings on the preference for wider corridors during the evacuation process^10,40^ and further promotes the ongoing discussion on the design of aided evacuation routes in buildings, which should be considered to lead through wide rather than narrow corridors to facilitate the evacuation process. Also, respondents tended to choose shorter rather than longer corridors. Respondents did not differentiate between medium and long corridors in this study; the crucial difference observed was that the shortest corridors were chosen more often. These results were expected and correspond to the previous observations^57^. Also, the data persuasively demonstrate that respondents consider the presence of stairs in the corridor as a very strong cue for an evacuation route since the likelihood of selecting a corridor with stairs was almost 10 times (or 15 times for the model with random slope terms for participants included) higher than selecting a corridor without stairs. The identified importance of staircases as an evacuation aid is further discussed below.

The absolute width and length of corridors are different in various buildings around the world and therefore, it is not entirely possible to generalize the conclusions reported above. Consequently, the second set of models considering a relative value—compactness of corridors—was estimated. Analysis showed that respondents tended to choose corridors with higher compactness. This is partially in contrast to the previous findings^34,36,37^, where higher compactness of the decision point induced higher complexity of decision-making processes. In these studies, however, the compactness was calculated for 360° isovists, whereas in our study, we assessed each corridor separately using partial isovists. Nevertheless, the fact that participants preferred short and wide corridors was expected correspondingly to the previous studies^10,40,57^ as mentioned above. Further, the respondents' preference for choosing corridors with stairs remained unchanged as well. As in the case of the previous calculation method, respondents were identified to prefer the variants with stairs almost ten times more often, which further underlines the significance of the staircase as a dominant building element in the evacuation process. Since this isovist metric can be easily calculated for all building corridors, our findings can be easily transferred and used in safety engineering and the construction industry. For example, an application in agent-based evacuation models seems promising, where the agent's choice of the evacuation route could be guided by the probability of choosing a corridor defined on the basis of its compactness.

In respect of the self-reported confidence about respondents' decisions, we observed that in the intersections containing a corridor with a staircase, respondents felt more confident about their choices. On the other hand, the lowest levels of confidence were reported in the tasks without a staircase. Also, respondents spent less time making their evacuation choice in intersections with stairs than in intersections without stairs. The clear preference for stairs was also reflected by the respondents in the closing questionnaire, where a psychological reflection of the situation fully corresponded with the respondents' behavioural reactions. Here, the respondents considered stairs as an unambiguous cue indicating successful evacuation. The following decreasing trend in the self-reported evaluation of width and length as an evacuation aid corresponded to behavioural responses as statistically analysed above. A certain level of conscious reflection of the decision-making process was present during the whole VR-based experiment, which questions the validity of VR measurements towards real-life evacuation scenarios where more spontaneous, intuitive, or emotional behaviour patterns would be expected^1,6,19,20^.

Based on this, we conclude that the stairs in the building, when present in the field of view, can be considered a very strong predictor of evacuation behaviour. This finding enhances the importance of staircases as a functional element in the building, as discussed in the previous studies^19,35,64,65^, regardless of their potential limitations during evacuation, such as the reported disorientation when using them^65,66^ or increased computational demands when navigating vertically^67^. They should be carefully considered when designing a building (e.g., differentiate their appearance from the surroundings and consider placement within the building matching the occupant's activity^65^) since they can have a significant effect on the evacuation process. The observation concerning the width and compactness of the corridors, respectively, further generates suggestions on the design of aided evacuation routes in buildings, which, in an effort to facilitate the evacuation process, should lead through wide and short corridors rather than long and narrow ones^7,10,22,40^.

This study has several limits which should be considered when making conclusions or designing follow-up research. First, despite the increasing quality of VR technologies and encouraging findings on the effectiveness of research of cognitive processes engaged in VR^10,44,45^, the main limitations of this study would be its virtual form, as well as web-based online data collection. Considering a certain level of conscious reflection of the decision-making process present during this whole experiment, the question of how evacuation behaviour might look in a real-world context persists. Also, the remote data collection format limits the reliability of the study since, despite detailed instructions, respondents could have used various HW/SW interfaces and workplaces to finish the tasks. The most unsatisfactory cases were filtered out based on the recorded information about the screen resolution used and whether the respondents worked in full-screen mode. Based on the high variance in reaction times, we can conclude that there are some suspicious careless responses present in the data analysis. However, the psychological scientific community does not currently agree on what is the threshold reaction time, from which the task solution process can already be considered valid.

Considering within subjects multifactorial design, participants given 20 tasks could have acquired a learned behaviour during the experimental session. We prevented this issue from occurring using standard techniques such as task order randomization and multiple outcome measures with delays using the Likert scale evaluation task as a filler*.* However, this prevention cannot be considered complete, and specific response preferences could have been learned among participants, which should bstated here as a limit of the study.

In the study, we decided to exclude any evacuation aids, as we wanted to limit the influence of this factor on decision-making since the participants could start noticing the signs only later in the course of the experiment. In real-world evacuations, it has been demonstrated that people often do not follow evacuation signs^17^, which may not be visible (due to inappropriate placement or the presence of smoke), and we wanted to analyse the respondents' behaviour in these cases. Also, in this study, the presented environment of the buildings was free of any decorative or other random elements in order for us to be able to draw direct conclusions about the influence of specific factors selected in the context of this study. Also, the lighting parameter was excluded in this study, which, as demonstrated by Vilar^40^, plays an important role in the selection of corridors. In a real-world scenario, such an environmental setting would probably not be possible, and the discussion concerning the ecological validity of VR-based experiments should continue further.

Since it was beyond the scope of this study, we would like to further address, for example, individual's current emotional states, cognitive and physical abilities, or situation and positional awareness as factors that can influence the decision-making process and behavioural preferences during the virtual evacuation. Even though this study explored general tendencies to evacuate with respect to selected building parameters, the influence of the awareness about the position in the building (e.g., occupying the specific floor) or potential physical disabilities of the evacuees (e.g., wheelchair users) should be among others further studied in the context of virtual-based experiments. Considering e.g., the scenario where participants would be aware that they evacuate themselves from the ground floor, the observed preference of the staircase as an evacuation hint would probably be decreased. The variety of behavioural responses should always be considered context-dependent, and follow-up research exploring specific space syntax and scenario settings is therefore needed.

In either case, we believe that the observations reported in the present study contribute to the very basis of the studied topic and will be further explored using various techniques with the aim to be potentially applied in engineering practice to prevent human or property threats in emergency evacuation scenarios.

## Supplementary Information

Supplementary Information 1.

Supplementary Figure 1.

Supplementary Table 1.

Supplementary Video 1.

## Data Availability

All datasets generated during and/or analysed during the current study are available from the corresponding author on reasonable request.
