# Supplementary material for: Isovists compactness and stairs as predictors of evacuation route choice
Source: Sci Rep. 2023 Feb 20;13:2970. doi: 10.1038/s41598-023-29944-8 (PMC9940685; doi:10.1038/s41598-023-29944-8)
Supplement: Supplementary file 1 — Supplementary Information 1. [file 41598_2023_29944_MOESM1_ESM.docx]

**Supplementary Figure:** Schematic overview of all T-intersection layouts from the Task Stimuli database. *Encoding: Width – W represents wide corridors of 4 m width, N represents corridors of 2 m width; Length – S represents short corridors of 10 m length, M represents middle corridors of 15 m length, L represents long corridors of 20 m length; Stairs – 0 represents corridors without stairs, 1 represents corridors with stairs.*
