# Supplementary material for: Isovists compactness and stairs as predictors of evacuation route choice
Source: Sci Rep. 2023 Feb 20;13:2970. doi: 10.1038/s41598-023-29944-8 (PMC9940685; doi:10.1038/s41598-023-29944-8)
Supplement: Supplementary file 3 — Supplementary Table 1. [file 41598_2023_29944_MOESM3_ESM.docx]

**Supplementary Table 1.** Distribution of corridor choices, reaction time, and level of confidence

|  | **presence of stairs** | **corridor width** | **corridor length** | **corridor compactness** | **number of selected** | **number of not selected** | **selection rate [%]** | **reaction time (s)** | | | | | **confidence (1 - very unsure,  5 - very confident)** | | | |
| --- | --- | --- | --- | --- | --- | --- | --- | --- | --- | --- | --- | --- | --- | --- | --- | --- |
|  |  |  |  |  |  |  |  | **Mean** | **Median** | **Min** | **Max** | **Sd** | | **Mean** | **Median** | **Sd** |
| **tasks with stairs** | 0 | 2 | 10 | 0.37 | 57 | 478 | 10.65 | 3.50 | 3.48 | 1.12 | 7.57 | 1.40 | | 3.49 | 4 | 0.95 |
|  | 0 | 2 | 15 | 0.30 | 62 | 748 | 7.65 | 3.79 | 3.76 | 0.86 | 7.57 | 1.42 | | 3.26 | 3 | 0.89 |
|  | 0 | 2 | 20 | 0.25 | 33 | 473 | 6.52 | 3.55 | 3.10 | 1.60 | 7.91 | 1.45 | | 3.12 | 3 | 1.05 |
|  | 0 | 4 | 10 | 0.66 | 66 | 195 | 25.29 | 3.80 | 3.57 | 0.98 | 7.41 | 1.56 | | 3.35 | 3 | 1.00 |
|  | 0 | 4 | 15 | 0.54 | 72 | 340 | 17.48 | 4.04 | 3.82 | 1.02 | 7.64 | 1.62 | | 3.33 | 3 | 1.07 |
|  | 0 | 4 | 20 | 0.46 | 41 | 196 | 17.30 | 4.05 | 3.60 | 1.44 | 7.84 | 1.79 | | 3.22 | 3 | 0.94 |
|  | 1 | 2 | 10 | 0.37 | 440 | 69 | 86.44 | 3.29 | 3.04 | 0.80 | 7.35 | 1.20 | | 3.75 | 4 | 0.87 |
|  | 1 | 2 | 15 | 0.30 | 683 | 105 | 86.68 | 3.37 | 3.10 | 1.09 | 7.99 | 1.27 | | 3.73 | 4 | 0.84 |
|  | 1 | 2 | 20 | 0.25 | 441 | 94 | 82.43 | 3.61 | 3.36 | 1.11 | 7.76 | 1.30 | | 3.66 | 4 | 0.80 |
|  | 1 | 4 | 10 | 0.66 | 241 | 17 | 93.41 | 2.91 | 2.70 | 0.99 | 7.37 | 1.15 | | 4.12 | 4 | 0.77 |
|  | 1 | 4 | 15 | 0.54 | 382 | 30 | 92.72 | 3.18 | 2.93 | 1.08 | 7.30 | 1.20 | | 3.97 | 4 | 0.83 |
|  | 1 | 4 | 20 | 0.46 | 243 | 16 | 93.82 | 3.27 | 2.93 | 0.88 | 7.88 | 1.26 | | 3.98 | 4 | 0.86 |
| **tasks without stairs** | - | 2 | 10 | 0.37 | 261 | 248 | 51.28 | 3.97 | 3.85 | 0.91 | 7.99 | 1.41 | | 2.88 | 3 | 0.99 |
|  | - | 2 | 15 | 0.30 | 238 | 531 | 30.95 | 4.26 | 4.13 | 1.28 | 7.99 | 1.52 | | 2.61 | 3 | 0.91 |
|  | - | 2 | 20 | 0.25 | 107 | 401 | 21.06 | 4.24 | 3.85 | 1.45 | 7.60 | 1.65 | | 2.65 | 3 | 1.01 |
|  | - | 4 | 10 | 0.66 | 230 | 16 | 93.50 | 3.55 | 3.14 | 1.39 | 7.45 | 1.31 | | 3.12 | 3 | 1.01 |
|  | - | 4 | 15 | 0.54 | 325 | 77 | 80.85 | 3.65 | 3.44 | 1.27 | 7.93 | 1.37 | | 3.04 | 3 | 0.96 |
|  | - | 4 | 20 | 0.46 | 191 | 79 | 70.74 | 3.68 | 3.34 | 1.48 | 7.78 | 1.36 | | 3.15 | 3 | 0.90 |
